# Outcomes of lumbar spinal fusion in super-elderly patients aged 80 years and over: Comparison with patients aged 65 years and over, and under 80 years

**DOI:** 10.1097/MD.0000000000026812

**Published:** 2021-08-06

**Authors:** Hee Jung Son, Young-Hoon Jo, Hyung Seob Ahn, Jooyoung You, Chang-Nam Kang

**Affiliations:** a Department of Orthopedic Surgery, Hanyang University College of Medicine, Seoul, Korea; b Department of Orthopedic Surgery, Hanyang University, College of Medicine, Guri Hospital, Gyeonggi, Korea.

**Keywords:** 80 and over, complications, lumbar spinal fusion, super-elderly, surgical outcome

## Abstract

Despite the increasing prevalence of spinal surgery in super-elderly (SE) patients, the outcomes and complication rates have not been fully elucidated. The purpose of this study was to compare the outcomes and complications of lumbar spinal fusion for degenerative lumbar spinal stenosis (DLSS) in SE patients aged 80 years and over with those in patients aged 65 years and over, and under 80 years.

This study analyzed 160 patients who underwent spinal fusion for DLSS between January 2011 and November 2019. Thirty patients in the SE group (group SE, ≥80 years) and 130 patients in the elderly group (group E, ≥65 years and <80 years) were enrolled. The performance status was evaluated by preoperative American society of anesthesiologists (ASA) score. Visual analog scales for back pain (VAS-BP) and leg pain (VAS-LP), and Korean Oswestry disability index (K-ODI) were used to assess clinical outcomes preoperatively and 1 year postoperatively. Percent changes of VAS-BP, VAS-LP and K-ODI were also analyzed. Fusion rates were evaluated by computed tomography 6 months and 1 year postoperatively. Furthermore, bone mineral density, operative time, estimated blood loss, blood transfusion, hospital days, hospitalization in intensive care unit and postoperative complications were compared.

The average age of group SE was 82.0 years and that of group E was 71.6 years. There were no differences in preoperative ASA score, preoperative or postoperative VAS BP and VAS-LP, bone mineral density, operative time, estimated blood loss, blood transfusion, hospital days, hospitalization in intensive care unit and fusion rates between the groups. Preoperative and postoperative K-ODI were higher in group SE than group E (all *P* < .05). However, percent changes of VAS-BP, VAS-LP and K-ODI showed no significant differences. Overall early and late complications were not significantly different between the groups; however postoperative delirium was more common in group SE than group E (*P* = .027). SE status was the only risk factor for postoperative delirium with odds ratio of 3.4 (*P* = .018).

Spinal fusion surgery is considerable treatment to improve the quality of life of SE patients with DLSS, however careful perioperative management is needed to prevent postoperative delirium.

## 1 Introduction

According to statistics published by the United States Census Bureau in 2016, people aged 65 years and older accounted for 8.5% of the world's population in 2015, and this is expected to increase to 16.7% by 2050. The population aged 80 and over will more than triple between 2015 and 2050, from 126.5 million to 446.6 million. Furthermore, global life expectancy at birth is projected to increase from 68.6 years in 2015 to 76.2 in 2050. Even in Japan, life expectancy at birth was 84.7 years in 2015 and is expected to reach 91.6 years in 2050.^[1]^

As the aging population increases, degenerative spinal diseases such as degenerative spinal stenosis, degenerative disc disorder, and adult spinal deformity also increase.^[2,3]^ In the field of orthopedics, the term “super-elderly” (SE) refers to patients of 80 and older.^[4]^ SE patients with degenerative spinal disease experience difficulties in their daily life due to low back pain and leg pain, and they complain of their poor quality of life.^[5,6]^ Although medication, physical therapy and epidural steroid injections are used to manage those patients, some of such patients complain of debilitating persistent pain and functional disability. In these patients, appropriate surgical treatment should be considered.^[5,7]^ More aggressive treatment such as decompression or spinal fusion surgery may be necessary because many elderly patients remain socio-physically active as life expectancy increases.

There are several reports of favorable clinical and radiological outcomes of spinal fusion for elderly patients.^[8–10]^ However, the surgical outcomes in SE patients are not fully documented. The purpose of this study was to compare the outcomes and complications of lumbar spinal fusion for degenerative lumbar spinal stenosis (DLSS) in SE patients aged 80 years and over with those in patients aged 65 years and over, and under 80 years.

## 2 Materials and methods

### 2.1 Patient enrollment

This was a retrospective study approved by the institutional review board (HY-IRB: 2020–12–019–001). Patients who underwent spinal fusion surgery for DLSS between January 2011 and November 2019 and had at least 1 year of follow-up were eligible for inclusion. Surgery was performed on patients who reported severe persisting radiating pain to a lower extremity, neurogenic claudication no more than 100 meters, and/or low back pain despite conservative treatment lasting at least 3 months, and /or who had a neurologic deficit. All the patients had severe spinal stenosis as indicated by Schizas grades C or D, or foraminal stenosis of grade 3 on magnetic resonance imaging.^[11–13]^ Patients who received spinal fusion due to spinal trauma, tumor, deformity, infection or revision surgery were excluded.

The sample size was calculated referencing a previous study in consultation with medical statistics support office of our institution.^[9]^ It was set to obtain a power of 80% with an alpha of 0.05, thus, 160 patients were required. We classified patients of 80 years and older as the SE group (Group SE), those 65 and older, and under 80 as the elderly group (Group E).

Operation time, estimated blood loss, blood transfusion, hospital days and whether hospitalized in an intensive care unit were also analyzed. Bone mineral density was examined by dual-energy X-ray absorptiometry. Preoperative assessment included American society of anesthesiologists (ASA) score on physical status and medical comorbidities such as hypertension, diabetes mellitus (DM), coronary artery disease, arrhythmia, congestive heart failure, chronic kidney disease, asthma, chronic respiratory disease, history of tuberculosis, history of pulmonary thromboembolism, hepatitis, hyper- and hypothyroidism, dementia, history of stroke, Parkinson's disease and autoimmune disease.^[14]^

### 2.2 Surgical procedures

All surgical procedures were performed by 1 senior spine surgeon (CNK) at a single center. A posterior midline approach under general anesthesia was done. Decompressive laminectomy and pedicle screw fixation were performed. Cement-augmented cannulated pedicle screws were placed in the case of patients with 2 or more risk factors for implant failure among osteoporosis, >65 years of age, autoimmune disease and stage 3 to 5 chronic kidney disease, while solid pedicle screws were used for those without risk factors. In patients with only one of the 4 risk factors, selection of the screws was made by the surgeon, considering the general condition of the patients.^[15]^ After facetectomy and discectomy of the involved segments, open transforaminal lumbar interbody fusion was performed using 2 polyetheretherketone cages filled with morselised laminar bone and demineralized bone matrix.

### 2.3 Clinical and radiological outcomes

Clinical and functional outcomes were evaluated using preoperative and postoperative visual analog scales for back pain (VAS-BP) and leg pain (VAS-LP) and the Korean Oswestry disability index (K-ODI). Postoperative outcomes were measured 1 year postoperatively. In addition, percent changes of VAS-BP, VAS-LP and K-ODI were evaluated. Percent change were calculated as (postoperative value-preoperative value) / preoperative value × 100 (%).

For radiological outcomes, standing anteroposterior and lateral plain radiographs were obtained 1 week, 6 weeks, 3 months, 6 months, and 1 year after surgery. The Brantigan, Steffee and Fraser (BSF) classification was used to confirm interbody fusion grade, based on computed tomography at 6 months and 1 year after surgery.^[16]^ BSF-1 and 2 were classified as nonunion, BSF-3 as union. All radiographic assessment was performed independently by a spine fellow (HJS) and chief orthopedic resident (HSA), who did not participate in the surgery.

### 2.4 Postoperative complications

Medical records were reviewed to investigate postoperative complications. Complications were classified as early or late according to time of occurrence based on findings at postoperative 3 months. Early complications included major and minor complications. Major complications were death, neurologic deficit, leakage of cerebrospinal fluid, deep wound infection, congestive heart failure, deep vein thrombosis, pulmonary thromboembolism, acute myocardial infarction/ischemia, pneumonia, atelectasis, acute kidney injury and stroke. Minor complications included wound dehiscence, superficial wound infection, hematoma, urinary tract infection, urinary disturbance, ileus, gastritis, ischemic colitis and postoperative delirium.^[17,18]^ Late complications included adjacent segment disease, revision surgery and implant failure. All implant failures were defined as a radiolucent line over 2 mm between bone and implant or a disruption of the continuity of implant in the plain radiographs or computed tomography.

### 2.5 Statistical analysis

Statistical analysis was performed using SPSS 18.0 (SPSS Inc., Chicago, IL). Student *t* test or the Mann–Whitney test was used for continuous variables, and Chi-Squared or Fisher exact tests for categorical variables. Multivariate logistic regression analysis to investigate risk factors of postoperative complications was carried out with variables with *P* < .1 in the univariate analysis. Differences were considered statistically significant at *P* < .05.

## 3 Results

A total of 160 patients were enrolled with 30 and 130 patients assigned to group SE and group E, respectively. The average age was 82.0 years for group SE, and 71.6 years for group E. Demographic data did not differ between the 2 groups except for age and body mass index. Operative time, estimated blood loss, blood transfusion, hospital days and intensive care unit hospitalization also did not differ (Table 1). And there was no difference in the frequency of use of cement-augmented cannulated pedicle screws (*P* = .473). The proportion of patients with 1 or more comorbidities was similar and mean preoperative ASA scores were not different in the 2 groups, indicating that the groups were of much the same preoperative physical status (Table 2).

**Table 1:** Demographics and baseline characteristics.

	Group SE (n = 30)	Group E (n = 130)	*P*
Age at surgery (yr)	82.0 ± 2.1	71.6 ± 4.2	.000^∗^
Gender (male/female)	12/18	32/98	.089
^1^BMI (kg/m^2^)	22.8 ± 3.4	24.3 ± 3.6	.044^∗^
Osteoporosis	16 (53.3%)	48 (36.9%)	.098
^2^BMD (T-score)	−2.4 ± 1.1	−2.2 ± 1.0	.318
Preoperative ^3^Hb (g/dL)	12.2 ± 1.1	12.6 ± 1.5	.103
Preoperative albumin (g/dL)	3.9 ± 0.4	4.1 ± 0.4	.094
Number of fused segments	2.1 ± 1.4	2.2 ± 1.4	.869
Operative time (minutes)	339.5 ± 97.3	337.6 ± 93.2	.921
^4^EBL (mL)	1285.0 ± 1018.6	1208.7 ± 868.2	.675
Blood transfusion (mL)	1031.3 ± 773.7	786.2 ± 955.1	.192
Hospital days	33.1 ± 18.3	27.5 ± 25.8	.260
^5^ICU hospitalization	2 (6.7%)	1 (0.8%)	.090
Follow-up period (mo)	33.7 ± 22.4	28.0 ± 18.4	.146

**Table 2:** Preoperative assessment.

	Group SE (n = 30)	Group E (n = 130)	*P*
^1^ASA score			.253
ASA 1	0	0	
ASA 2	17 (56.7%)	94 (72.3%)	
ASA 3	13 (43.3%)	35 (26.9%)	
ASA 4	0	1 (0.8%)	
ASA 5	0	0	
Mean ASA score	2.43 ± 0.50	2.28 ± 0.47	.125
Number of patients with one or more comorbidities	28 (93.3%)	103 (79.2%)	.071

### 3.1 Clinical and radiological outcomes

There were no significant differences between the 2 groups in preoperative or postoperative VAS-BP and VAS-LP. On the other hand, preoperative and postoperative K-ODI was significantly higher in group SE than group E (*P* = .017, 0.022, respectively). However, there was no difference was observed in percent change of VAS-BP, VAS-LP or K-ODI. Fusion rates at 6 months were 70.0% in group SE and 68.5% in group E, and at 1 year 90.0% in group SE and 90.8% in group E; they were not different significantly (Table 3).

**Table 3:** Clinical and radiological outcomes.

	Group SE (n = 30)	Group E (n = 130)	*P*
Preoperative ^1^VAS-BP	6.2 ± 1.7	5.5 ± 2.4	.076
Preoperative ^2^VAS-LP	6.1 ± 1.7	6.5 ± 2.1	.312
Preoperative ^3^K-ODI	31.3 ± 5.5	28.2 ± 6.6	.017^∗^
Postoperative VAS-BP	2.8 ± 1.7	2.2 ± 1.8	.125
Postoperative VAS-LP	2.4 ± 1.7	2.3 ± 1.8	.597
Postoperative K-ODI	18.1 ± 6.3	14.7 ± 7.7	.022^∗^
Percent change of VAS-BP	−53.1 ± 32.1	−57.6 ± 32.6	.767
Percent change of VAS-LP	−58.4 ± 27.6	−65.7 ± 26.8	.183
Percent change of K-ODI	−41.8 ± 18.2	−48.3 ± 25.5	.108
Fusion rates_6 mo	21 (70.0%)	89 (68.5%)	.870
Fusion rates_1 y	27 (90.0%)	118 (90.8%)	1.000

### 3.2 Postoperative complications

No patients died from surgery-related complications during either hospital stays or follow-up. Overall early and late complications were not different between the 2 groups. However, there were significant differences in details. Unlike rates of major complications, minor complications rates were significantly different (16.7% vs 16.9%, *P* = .973 and 53.3% vs 32.3%, *P* = .031, respectively). Among the minor complications, only postoperative delirium was significantly higher in group SE than group E (26.7% vs 9.2%, *P* = .027) (Table 4). In multivariate logistic regression analysis, blood transfusion was a significant risk factor for major complications (*P* = .015), and SE status for delirium (*P* = .018), with a predictabilities of 84.4% and 88.1%, respectively (Table 5).

**Table 4:** Postoperative complications.

	Group SE (n = 30)	Group E (n = 130)	*P*
Early complications	17 (56.7%)	57 (43.8%)	.204
Major complications	5 (16.7%)	22 (16.9%)	.973
Neurologic deficit	1 (3.3%)	5 (3.8%)	1.000
Leakage of ^1^CSF	1 (3.3%)	1 (0.8%)	.341
Deep wound infection	2 (6.7%)	7 (5.4%)	.676
^2^CHF	0	0	-
^3^DVT	0 (0%)	1 (0.8%)	1.000
^4^PTE	0 (0%)	1 (0.8%)	1.000
^5^AMI	1 (3.3%)	6 (4.6%)	1.000
Pneumonia	1 (3.3%)	1 (0.8%)	.341
Atelectasis	0 (0%)	2 (1.6%)	1.000
^6^AKI	0 (0%)	1 (0.8%)	1.000
Stroke	0 (0%)	1 (0.8%)	1.000
Minor complications	16 (53.3%)	42 (32.3%)	.031^∗^
Wound dehiscence	0 (0%)	4 (3.1%)	1.000
Superficial wound infection	1 (3.3%)	6 (4.6%)	1.000
Hematoma	1 (3.3%)	1 (0.8%)	.341
^7^UTI	5 (16.7%)	14 (10.8%)	.358
Urinary disturbance	6 (20.0%)	11 (8.5%)	.094
Ileus	0 (0%)	4 (3.1%)	1.000
Gastritis	0 (0%)	3 (2.3%)	1.000
Ischemic colitis	0 (0%)	1 (0.8%)	1.000
Postoperative delirium	8 (26.7%)	12 (9.2%)	.027^∗^
Late complications	13 (43.3%)	56 (43.1%)	.980
Implant failure^†^	11 (36.7%)	53 (40.8%)	.679
^8^ASD	2 (6.7%)	6 (4.6%)	.645
Revision surgery	2 (6.7%)	7 (5.4%)	.676
Total	24 (80.0%)	87 (66.9%)	.161

**Table 5:** Multivariate analysis of risk factors for postoperative complications.

	Odds ratio (^1^CI)	*P*
Blood transfusion^†^	1.001 (1.000–1.001)	.015^∗^
Super-elderly^‡^	3.398 (1.233–9.367)	.018^∗^

## 4 Discussion

Some SE patients with DLSS hesitate to undergo surgical treatment because of concerns about their physical status and comorbidities, and this makes them prefer continuing conservative treatment.^[17,19]^ There is still controversy over which treatment is more appropriate for improving the quality of life of SE patients. Katz et al^[20]^ reported that satisfaction rates for surgical treatment of spinal fusion were lower in patients of poorer physical status or with more comorbidities. Deyo et al^[21]^ reported that the rates of complications after lumbar spinal surgery in patients aged 75 years or older reached 18%. And Johnsson et al^[22]^ recommended that elderly patients with DLSS should consider conservative treatment rather than surgery because few patients developed serious conditions over 4 years of observation.

However, even patients with more than 1 comorbidity have relatively good clinical outcomes after spinal fusion.^[8–10]^ According to Cho et al,^[9]^ clinical outcomes and complication rates following lumbar fusion surgery for DLSS did not differ between patients aged 75 and over, who had higher preoperative ASA scores, and those aged 65 years and over, and under 75 years. Also Ragab et al^[8]^ reported that clinical outcomes in 118 patients of 70 and over with several comorbidities who underwent decompressive surgery of the lumbar spine were similar to those of a younger group. In that study, 21 of the patients were older than 80 and no 1 died either in hospital or soon after surgery.

When performing spinal surgery in SE patients, spine surgeons are most concerned about postoperative complications because several studies have reported that these complications are higher in older patients. In the present study, 16.9% of all the patients (27 of a total of 160 patients) suffered major complications, a rate similar to those in other studies.^[19,21,23]^ However, there is no consensus about whether age by itself is a risk factor for postoperative complications after spinal surgery in elderly patients. Nasser et al^[24]^ reported that age increased the risk of complications after surgery for degenerative spondylosis among 79,417 patients. According to Carreon et al,^[17]^ when posterior lumbar decompression and fusion were performed in patients 65 and older, complication rates increased not only with age but with increased blood loss, longer operative time and number of fusion levels. In contrast, other studies reported that increasing numbers of comorbidities, higher BMI, DM and malnutrition, but not advanced age, were risk factors for perioperative complications after spinal surgery.^[8,25–27]^

In the present study, rates of early and late complications were not significantly different between the 2 groups. Although blood transfusion was a risk factor for early major complications, the odds ratio of 1.001 was too low to justify application to clinical situations. Therefore, it will be necessary to evaluate the risk factors for complications after spinal fusion surgery in SE patients in longer follow-up studies with higher levels of evidence in larger patient groups. Otherwise, postoperative delirium was significantly more frequent in the SE group. SE status was the only risk factor for postoperative delirium, with an odds ratio of 3.4. Similarly, Song et al^[28]^ found that postoperative delirium was more common after orthopedic surgery in older patients, especially after spine, hip and knee surgery; frequencies were 1.18% in the 50 s, 3.86% in the 60 s, 8.49% in 70 s, and 13.04% in the over 80 s. Therefore, spine surgeons should focus on nonpharmacological and pharmacological methods for preventing postoperative delirium when performing spinal fusion surgery in SE patients.^[29,30]^ Rates of implant failure in the present study were 36.7% and 40.8% in group SE and group E, respectively. It was lower than 62.8%, screw loosening rates for osteoporotic vertebra reported by El Saman et al,^[31]^ however it was relatively high. It might be due to strict criteria of implant failure over 2 mm. Recently, the authors use cement-augmented cannulated pedicle screws to prevent implant failure.^[15]^

There was no significant difference between the 2 groups in preoperative or postoperative VAS-BP and VAS-LP in the present study. In addition, percent changes of VAS-BP and VAS-LP were also not different. In a previous study, there was a tendency for average ODI to increase gradually from 20 s to 70 s.^[32]^ Likewise, the preoperative and postoperative K-ODI was significantly higher in the SE patients than the elderly patients. However, there was no difference in percent change of K-ODI and percent change of ODI is known to be the best marker of outcome when such subjective scoring systems are used in lumbar spinal surgery.^[33]^ These results showed that satisfactory clinical and functional outcomes were obtained after spinal fusion surgery for DLSS in SE patients in the present study.

Our study has a number of notable limitations. First, it was a retrospective study of spinal fusion surgery performed by 1 surgeon in a single center. Second, the SE group was relatively small due to the paucity of the patients in that age distribution. Third, the follow-up period of a minimum of 1 year was too short to evaluate late complications such as adjacent segment disease and revision surgery. However, because early major complications of spinal surgery are of more concern in SE patients, late complications may not be critical. Lastly, there might have been selection bias because SE patients in a very poor physical state or with a lot of severe comorbidities might not have undergone surgery.

## 5 Conclusions

Spinal fusion surgery for DLSS in SE patients resulted in more minor complications, especially postoperative delirium, than in elderly patients, however similar rates of early major complications, late complications, improvements of clinical and functional outcomes, and fusion rates. The only risk factor for postoperative delirium in this study was SE status, with an odds ratio of 3.4. Therefore, spinal fusion surgery is considerable treatment to improve the quality of life of SE patients with DLSS, however careful perioperative management is needed to prevent postoperative delirium.

## Author contributions

**Conceptualization:** Hee Jung Son, Chang-Nam Kang.

**Data curation:** Hyung Seob Ahn, Jooyoung You.

**Formal analysis:** Hyung Seob Ahn, Jooyoung You.

**Supervision:** Chang-Nam Kang.

**Writing – original draft:** Hee Jung Son, Young-Hoon Jo.

**Writing – review & editing:** Hee Jung Son, Young-Hoon Jo, Chang-Nam Kang.
